# Navigating the immunosuppressive abyss: current hurdles and innovative breakthroughs in neoadjuvant immunotherapy for osteosarcoma

**DOI:** 10.3389/fimmu.2026.1837053

**Published:** 2026-09-14

**Authors:** Guanghao Chen, Minjie Hu, Zhongkun Li, Kunpeng Yang, Bao Chen, Tianhao Xu

**Affiliations:** 1 The Second Hospital of Jiaxing, Department of Orthopaedics, The Second Affiliated Hospital of Jiaxing University, Jiaxing, China; 2 The Third Department of Medical Oncology, Jilin Cancer Hospital, Changchun, Jilin, China; 3 Department of Thoracic Oncology Surgery II, Jilin Cancer Hospital, Changchun, Jilin, China

**Keywords:** genomic instability, natural killer cells, neoadjuvant immunotherapy, osteosarcoma, pathological response assessment, tumor immune microenvironment, tumor-draining lymph nodes

## Abstract

Osteosarcoma is the most common primary malignant bone tumor of children and adolescents. Multiagent neoadjuvant chemotherapy once improved survival substantially, but outcomes have since plateaued. Delivering immunotherapy before surgery is attractive because the primary tumor, the tumor immune microenvironment (TIME), and the tumor-draining lymph nodes (TDLNs) remain intact. This setting may allow the TIME to be reconfigured while antigen sampling and T-cell priming are still possible. It does not, however, make the tumor a vaccine, nor does it imply that expanded tumor-infiltrating lymphocytes are tumor specific. Any benefit is instead likely to depend on antigen quality, human leukocyte antigen (HLA)-mediated presentation, the availability of tumor-reactive T-cell clones, and the reversibility of local immunosuppression. Translation remains constrained by a low but heterogeneous mutational burden, a myeloid-dominated microenvironment, and the absence of validated selection biomarkers. Preoperative treatment must also preserve its established non-immunological goals, namely downstaging to enable limb salvage and control of micrometastatic disease. We therefore examine the biological rationale, the current evidence, and the surgical, radiological, and pathological barriers to translation. We additionally consider how structural genomic instability constrains antigen availability and presentation, and how natural killer cell-directed strategies may inform barrier-matched design. On this basis we propose a barrier-matched development framework in which pathological response, spatial multi-omics, and molecular clearance jointly define success.

## 1 Introduction

### 1.1 Historical evolution and the survival plateau in osteosarcoma

Osteosarcoma is a highly aggressive mesenchymal malignancy that predominantly arises in the metaphyseal regions of long bones in children and adolescents (1, 2). Over the past four decades, its management has undergone a major transformation. Before the introduction of chemotherapy, treatment relied primarily on surgical amputation, and long-term outcome was poor. In a randomized trial with concurrent controls, patients treated with surgery alone had a two-year relapse-free survival of 17%, similar to that reported in studies conducted before 1970, and a projected six-year event-free survival of 11% (3, 4). The subsequent introduction of the MAP regimen, consisting of high-dose methotrexate, doxorubicin, and cisplatin, established a multimodal standard of care. The integration of preoperative neoadjuvant chemotherapy (NACT), surgical resection, and postoperative adjuvant chemotherapy increased the 5-year survival rate to approximately 60–70% (1, 5–7). In addition to improving survival, this treatment paradigm made limb-salvage surgery feasible for many patients, thereby substantially improving their quality of life.

Despite these advances, improvements in clinical outcomes have subsequently plateaued. Over the past three decades, clinical trials have evaluated dose intensification, alternative administration strategies, and the incorporation of additional cytotoxic agents, including ifosfamide and etoposide. Nevertheless, overall survival in osteosarcoma has remained largely unchanged (8). The landmark international EURAMOS-1 randomized controlled trial further demonstrated that, among patients with a poor histological response to NACT, the addition of ifosfamide and etoposide to postoperative chemotherapy did not improve event-free survival and increased toxicity (9). This high-level evidence suggests that chemotherapy regimens based on conventional DNA-damaging mechanisms may have approached the limits of acceptable toxicity and achievable clinical benefit. Immunotherapy is not, however, an untested proposition in this disease. In a randomized phase 3 trial conducted by the Children’s Oncology Group in 662 patients with resectable nonmetastatic osteosarcoma, the addition of muramyl tripeptide, a macrophage-activating agent, to standard chemotherapy improved 6-year overall survival from 70% to 78% (*P* = 0.03; hazard ratio 0.71, 95% CI 0.52–0.96), with a trend toward improved event-free survival (*P* = 0.08) (10). Two qualifications matter for the argument developed in this review. The agent acts through innate myeloid activation rather than through the adaptive, antigen-specific mechanisms on which checkpoint blockade depends, so the result does not by itself predict benefit from T-cell-directed strategies. It also remains, nearly two decades later, the only positive randomized phase 3 immunotherapy result in osteosarcoma. Read together with the failure of cytotoxic intensification, however, it establishes that this tumor is not categorically refractory to immunological intervention, and that the relevant question is which immune barrier a given strategy is intended to overcome. Therefore, there is an urgent need to investigate novel systemic treatments with non–cross-resistant mechanisms, particularly immunotherapeutic strategies capable of remodeling the antitumor immune microenvironment (7).

The prognosis remains poor for patients who present with metastatic disease at diagnosis, respond inadequately to chemotherapy, or develop recurrent disease after treatment, with 5-year survival rates below 30% (11, 12). Outcomes are particularly unfavorable for the approximately one in ten patients who present with clinically detectable metastases, most commonly pulmonary (11), and for those with a poor histological response to NACT, conventionally defined by a tumor necrosis rate (TNR) below 90% or, equivalently, more than 10% residual viable tumor (8, 9). As discussed below in Section 4.2, TNR is a historical osteosarcoma-specific term, and contemporary pathological assessment frameworks instead report the percentage of residual viable tumor (%RVT).

### 1.2 Rationale for neoadjuvant immunotherapy

Immunotherapy, particularly immune checkpoint inhibitors (ICIs) targeting programmed cell death protein 1 (PD-1) or its ligand, programmed death-ligand 1 (PD-L1), has achieved substantial clinical success in several malignancies, including melanoma, non-small cell lung cancer (NSCLC), and colorectal cancer (13–15). Administering immunotherapy in the neoadjuvant setting, before surgical resection, may offer several theoretical advantages.

Unlike adjuvant treatment, which is administered after tumor removal, neoadjuvant therapy is delivered while the primary tumor and tumor-draining lymph nodes (TDLNs) remain anatomically intact. Tumor mutational burden (TMB) is a genomic characteristic rather than a direct measure of tumor volume. Accordingly, a greater tumor burden does not necessarily correspond to a larger repertoire of antigens that are expressed, processed, and recognized by the immune system. Instead, the unresected primary tumor may serve as a continuous source of heterogeneous tumor-derived antigens, while preserved lymphatic drainage enables antigen-presenting cells to transport these antigens to the TDLNs. Thus, preservation of the tumor–lymphatic–TDLN axis may provide a favorable anatomical context for antigen sampling, dendritic cell trafficking, and the priming or expansion of potentially tumor-reactive T-cell clones (16, 17). As illustrated conceptually in **Figure 1**, the neoadjuvant setting preserves the primary tumor–lymphatic–TDLN axis and may maintain opportunities for antigen sampling and T-cell priming, whereas surgical resection may reduce, but does not necessarily eliminate, subsequent antigenic stimulation and immune activation.

**Figure 1:**
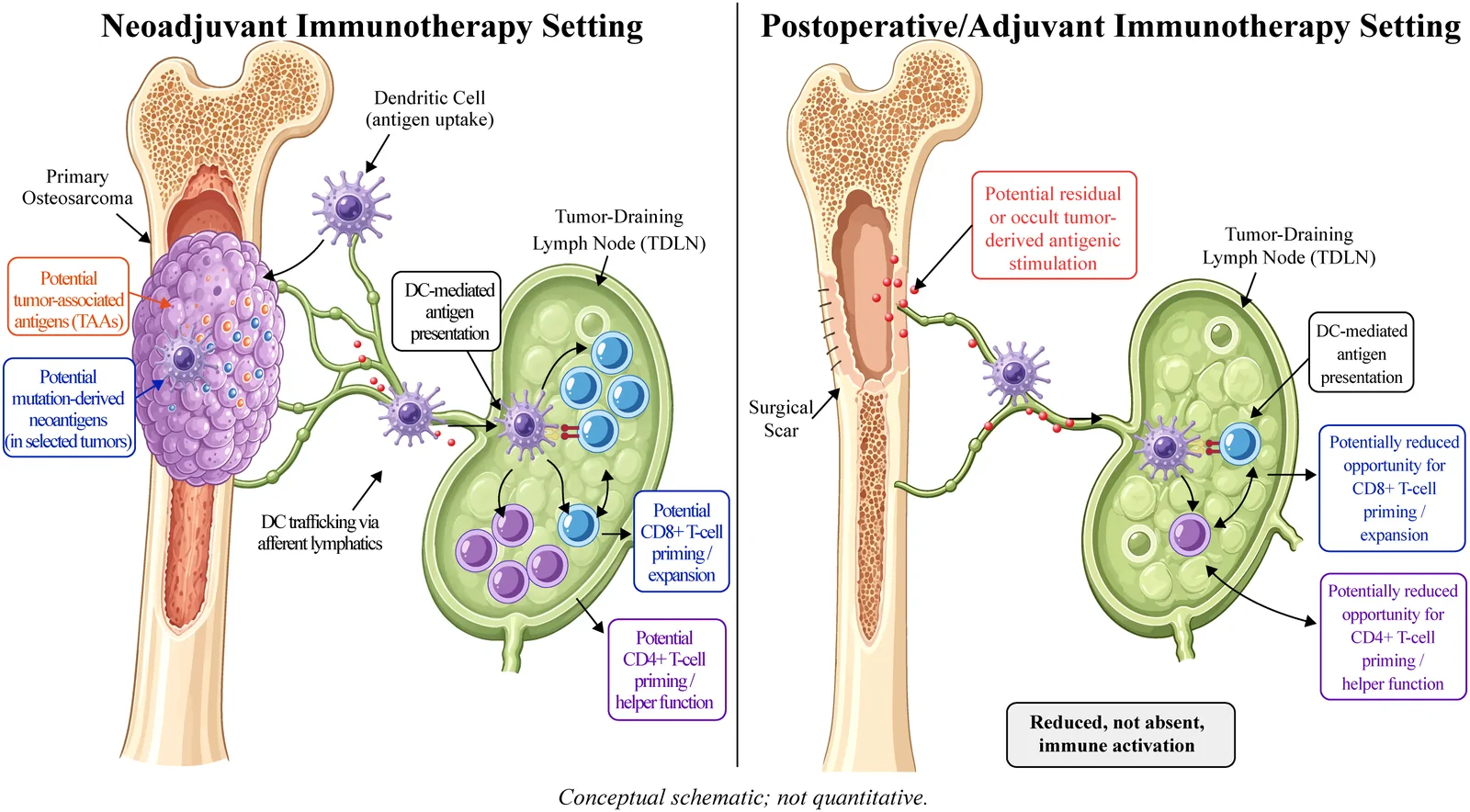
Conceptual comparison of the immunological contexts of neoadjuvant and postoperative/adjuvant immunotherapy in osteosarcoma. In the neoadjuvant setting, the primary osteosarcoma, afferent lymphatic drainage, and tumor-draining lymph nodes (TDLNs) remain anatomically intact. The unresected tumor may therefore provide a continuing source of heterogeneous tumor-derived antigens, including shared tumor-associated antigens (TAAs) and, in selected tumors, expressed mutation-derived neoantigens. Dendritic cells may capture and process these antigens, traffic via afferent lymphatic vessels, and present antigen-derived peptides to T cells within the TDLNs. This preserved tumor–lymphatic–TDLN axis may maintain opportunities for the priming, expansion, or functional activation of potentially tumor-reactive CD8+ T-cell clones and for CD4+ T-cell priming and helper activity. However, these processes depend on antigen expression and quality, intact antigen processing and HLA-mediated presentation, the availability of relevant T-cell clones, and the degree of local immunosuppression. Following surgical resection, the principal source of primary tumor-derived antigens is removed, and the associated lymphatic and TDLN interactions are altered. Nevertheless, antigenic stimulation is not necessarily eliminated because residual tumor cells, circulating or disseminated tumor cells, and occult micrometastatic lesions may persist. Dendritic-cell-mediated antigen presentation and T-cell activation may therefore continue after surgery, although the opportunity for *de novo* antigen sampling and T-cell priming may be reduced. The figure is a conceptual and non-quantitative schematic. It does not imply that the primary tumor functions as an *in situ* vaccine, that all tumor-infiltrating or expanded T cells are tumor reactive, or that neoadjuvant treatment invariably generates systemic antitumor immunity or durable immune memory. DC, dendritic cell; HLA, human leukocyte antigen; TAA, tumor-associated antigen; TDLN, tumor-draining lymph node.

The immunological rationale for neoadjuvant therapy should therefore be framed in terms of treatment-mediated reconfiguration of the tumor immune microenvironment (TIME), rather than an “*in situ* vaccine” effect. NACT, ICIs, and other immunomodulatory agents may modify antigen presentation, myeloid cell polarization, inhibitory signaling, and the recruitment and functional states of tumor-infiltrating lymphocytes (TILs) (16–19). In selected tumor contexts, these changes may facilitate the activation or reinvigoration of tumor-reactive T-cell clones and thereby promote systemic antitumor immunity. However, these effects are context dependent and should not be regarded as an inevitable consequence of retaining the primary tumor.

Importantly, the presence or numerical expansion of TILs should not be equated with tumor-specific reactivity. Studies of other human solid tumors have shown that tumor infiltrates may contain substantial populations of bystander CD8+ T cells that recognize viral or other non-tumor antigens. Moreover, the capacity of the intratumoral T-cell receptor repertoire to recognize autologous tumor antigens may be limited and variable (20, 21). Although these phenomena have not been specifically quantified in osteosarcoma, they caution against assuming that all lymphocytes present within osteosarcoma lesions, or expanded following treatment, are tumor reactive. Accordingly, the therapeutic objective should not be indiscriminate TIL expansion, but rather the recruitment, priming, and functional activation of T-cell clones capable of recognizing presented osteosarcoma antigens.

An intact primary tumor may expose the immune system to a broader repertoire of tumor-derived antigens than that remaining after surgical resection. These antigens may include shared tumor-associated antigens (TAAs) and, in selected tumors, expressed mutation-derived neoantigens. Nevertheless, the antigenic landscape of osteosarcoma is highly heterogeneous. Although osteosarcoma generally has a lower point-mutation burden than many malignancies responsive to ICIs, substantial interpatient and intratumoral heterogeneity exists in mutational burden, antigen expression, human leukocyte antigen (HLA) class I competence, and immune cell composition (22–25). Moreover, the extensive copy-number alterations and structural rearrangements characteristic of osteosarcoma do not necessarily generate neoantigens that are expressed and immunologically recognizable (24). Therefore, the primary tumor should be regarded as a potential source of heterogeneous tumor-derived antigens rather than as a uniformly abundant neoantigen reservoir. Any resulting immunological benefit is likely to depend on antigen quality and expression, intact antigen processing and HLA class I presentation, the availability of relevant T-cell clones, and the degree of immunosuppression within the TIME (18, 23, 24).

Surgical resection removes the principal source of primary tumor-derived antigens and disrupts the associated lymphatic and TDLN interactions. It may therefore reduce opportunities for continued antigen sampling and *de novo* T-cell priming (16, 17). However, resection does not necessarily eliminate all sources of antigenic stimulation, because residual tumor cells, circulating or disseminated tumor cells, and occult micrometastatic lesions may persist. Accordingly, the distinction between neoadjuvant and adjuvant treatment should be understood as a difference in immunological context rather than as the absolute presence or absence of tumor antigens.

Early control of micrometastatic disease is particularly important in osteosarcoma because pulmonary metastasis is the leading cause of disease-related mortality. Although many patients have no radiologically detectable metastases at diagnosis, subclinical micrometastatic disease may already be present. If neoadjuvant treatment successfully induces or expands tumor-reactive T-cell clones, these cells may recognize and control distant tumor deposits (17). However, this possibility remains a mechanistic hypothesis that requires prospective validation in osteosarcoma and should not be interpreted as an established systemic vaccination effect.

The neoadjuvant setting also provides a valuable window of opportunity for translational research. Comparisons between pretreatment biopsy specimens and post-treatment surgical specimens enable *in vivo* assessment of pharmacodynamic changes, antigen-presentation status, T-cell clonality, the spatial organization of immune cells, and remodeling of the TIME. Such paired-sample analyses may help distinguish the expansion of tumor-reactive lymphocytes from nonspecific or bystander immune-cell infiltration. They may also facilitate the identification of biomarkers associated with therapeutic response or resistance (13, 16, 21).

These considerations are particularly important in osteosarcoma. Although osteosarcoma is generally regarded as immunologically cold at the population level, it is biologically heterogeneous. TMB, antigen expression, HLA class I status, T-cell infiltration, myeloid cell composition, and the spatial organization of immune cells vary among patients, across different regions of the same tumor, and potentially between primary and metastatic lesions (22–25). Therefore, this review does not conceptualize the primary tumor as a vaccine or assume that neoadjuvant treatment inevitably induces durable protective immune memory. Instead, it examines whether preservation of the tumor–TDLN axis, together with treatment-mediated reconfiguration of the TIME, can promote biologically meaningful tumor-reactive immune responses in selected patients.

Accordingly, this review addresses a central translational question: Can biologically matched neoadjuvant immunotherapy induce a clinically meaningful pathological response in selected patients with osteosarcoma without compromising definitive surgical treatment? We first examine how preservation of the primary tumor–lymphatic–TDLN axis and treatment-induced reconfiguration of the TIME may provide an opportunity to activate antitumor immunity. We then distinguish evidence derived from advanced-disease salvage cohorts from evidence generated in genuine neoadjuvant settings involving prospectively planned surgery. On this basis, we characterize the antigen-presentation, microenvironmental, biomarker-related, surgical, and radiological barriers that may prevent immune activation from translating into effective tumor eradication. Finally, we propose a barrier-matched clinical development framework in which therapeutic strategies are evaluated according to pathological response, surgical feasibility, molecular clearance, and long-term oncological outcomes.

## 2 Biological rationale and current clinical evidence

The clinical development of neoadjuvant immunotherapy for osteosarcoma requires a careful alignment of biological plausibility with the strength of the currently available evidence. Although preservation of the tumor–lymphatic–TDLN axis provides a distinct immunological context, the available clinical evidence in osteosarcoma is derived predominantly from patients with advanced disease or from salvage-treatment cohorts. Accordingly, this section first examines the biological basis of preoperative immune modulation and then critically evaluates the conclusions that can, and cannot, be drawn from existing clinical studies.

### 2.1 Preservation of the tumor–TDLN axis and treatment-induced TIME remodeling

The osteosarcoma TIME is a complex ecosystem comprising malignant cells, infiltrating immune cells, stromal cells, the extracellular matrix, vascular structures, and soluble mediators (26). Although higher levels of CD4+ or CD8+ T-cell infiltration have been associated with favorable outcomes in observational cohorts (26), lymphocyte abundance alone does not establish tumor specificity or effective antitumor activity. Tumor infiltrates may contain bystander or virus-specific T cells, while tumor-reactive clones may constitute only a variable proportion of the intratumoral T-cell repertoire (20, 21).

Therefore, the potential advantage of the neoadjuvant setting does not arise from the assumption that the intact tumor functions as a vaccine. Rather, it lies in the opportunity to reconfigure the TIME while tumor-derived antigens, lymphatic drainage, and TDLNs remain available to support antigen sampling and T-cell priming. Whether this immunological context generates clinically meaningful antitumor immunity depends on antigen expression, intact HLA-mediated antigen presentation, the availability of tumor-reactive T-cell clones, and the extent to which local immunosuppression can be reversed (16–18, 23, 24).

This therapeutic opportunity is constrained by the myeloid-dominant immune landscape of osteosarcoma. M2-polarized tumor-associated macrophages, myeloid-derived suppressor cells, and regulatory T cells impair antitumor effector function through inhibitory cytokine signaling, immune checkpoint pathways, metabolic competition, and stromal exclusion (19, 27–31). Consequently, immune checkpoint blockade alone is unlikely to be effective unless treatment simultaneously addresses one or more of these nonredundant immunosuppressive barriers.

Conventional NACT may provide one mechanism for modifying this immunosuppressive environment. Chemotherapeutic agents can induce immunogenic cell death, promote the release of TAAs and damage-associated molecular patterns, and alter the composition of the immune microenvironment. In osteosarcoma, NACT has been associated with increased densities of CD3+ and CD8+ T cells, a higher abundance of PD-L1-positive immune cells, and a reduction in myeloid-derived suppressor cells (19). Similarly, multi-omic studies of non-small cell lung cancer have demonstrated enhanced antigen-presentation programs and alterations in B-cell and monocyte populations following neoadjuvant chemoimmunotherapy (32).

Collectively, these findings provide a biological rationale for combining chemotherapy with immunotherapy. However, they represent mechanistic and translational observations rather than definitive evidence of clinical benefit. The critical question, therefore, is whether existing osteosarcoma studies demonstrate that treatment-induced immune remodeling can translate into clinically meaningful tumor responses.

A final qualification is necessary before proceeding. The immunological framing developed above describes what may be distinctive about the neoadjuvant setting, not what makes preoperative treatment valuable in osteosarcoma. NACT was adopted, and remains standard, for two reasons that are largely independent of adaptive immunity: it reduces tumor volume and peritumoral reactive change, thereby defining a resection plane and making limb-salvage surgery feasible in most patients; and it treats occult micrometastatic disease at the earliest point at which systemic therapy can be delivered (5, 6). These cytoreductive and antimetastatic objectives remain the principal determinants of clinical benefit. Immune reconfiguration of the TIME should therefore be evaluated as an addition to these goals rather than as a replacement for them, and a regimen that reshapes the TIME while compromising local downstaging or delaying systemic control would not represent progress.

### 2.2 From monotherapy failure to combination strategies

A clear distinction must be made between salvage activity in advanced disease and efficacy within a true neoadjuvant treatment pathway. Most immunotherapy studies in osteosarcoma have enrolled patients with recurrent, refractory, or metastatic disease and have used objective response or progression-free survival as their principal endpoints. These studies generally did not incorporate prospectively planned surgical resection or standardized assessment of pathological response (33–38). Although these studies provide valuable information regarding therapeutic activity and mechanisms of resistance, they cannot independently establish the clinical benefit of neoadjuvant immunotherapy.

The phase 2 SARC028 trial illustrates the limited activity of single-agent PD-1 blockade in osteosarcoma. Among 22 evaluable patients with osteosarcoma, pembrolizumab induced only one objective response (5%) (34). Correlative analyses indicated that this exceptional responder exhibited a more immune-infiltrated phenotype at baseline, characterized by activated CD8+ T cells and PD-L1-positive macrophages (35). Similarly, KEYNOTE-051 reported no objective responses in its osteosarcoma cohort (36). In an independent phase 2 study (PROMO, NCT03013127) conducted by Boye et al., the median progression-free survival was 1.7 months, and the median overall survival was 6.6 months (33).

These findings indicate that PD-1 pathway inhibition alone is generally insufficient to overcome the multifaceted immune-evasion network of osteosarcoma. They further suggest that occasional responses are more likely to occur in biologically permissive tumors than in an unselected patient population. This observation supports biomarker-enriched trial designs rather than the indiscriminate application of immune checkpoint blockade to all patients.

Combination studies have yielded signals of greater antitumor activity but remain largely confined to the advanced-disease salvage setting. Lenvatinib plus pembrolizumab has demonstrated disease control in selected sarcoma cohorts, whereas apatinib plus camrelizumab achieved an objective response rate of 20.9% and a 6-month progression-free survival rate of 50.9% in patients with chemotherapy-refractory osteosarcoma (37, 38). Antiangiogenic agents may contribute to these effects by normalizing tumor vasculature and partially suppressing the activity of myeloid and regulatory immune cells. Nevertheless, these findings do not establish that such regimens improve pathological response, complete resection rates, or postoperative outcomes.

Collectively, the available studies demonstrate the feasibility and occasional antitumor activity of immunotherapy but do not show that it consistently induces deep pathological responses in resectable osteosarcoma. The discrepancy between the strong biological rationale and limited clinical activity highlights both the scarcity of evidence from true neoadjuvant settings and the barriers that prevent immune activation from translating into effective tumor eradication. The progression from single-agent checkpoint blockade to combination and barrier-matched strategies is summarized in **Supplementary Figure 1**, and selected representative clinical studies and their relevance to neoadjuvant pathological response are compared in **Table 1**.

**Table 1:** Selected representative clinical studies and their relevance to neoadjuvant pathological response in osteosarcoma.

Trial ID and study	Clinical phase and treatment setting	Core intervention and mechanism	Patient population	Key clinical outcomes or status	Translational or biomarker findings	Relevance to neoadjuvant pathological response
NCT02301039, SARC028	Phase II single-agent immunotherapy study conducted in the advanced-disease salvage setting.	Pembrolizumab; PD-1 pathway blockade.	Twenty-two evaluable patients with previously treated advanced osteosarcoma.	Only one objective response (5%) was observed among the 22 evaluable patients with osteosarcoma; the prespecified efficacy target was not met.	The exceptional responder exhibited a more immune-infiltrated baseline phenotype, including activated CD8+ T cells and PD-L1-positive macrophages, suggesting that pre-existing immune context may influence response.	Definitive surgery and standardized pathological response were not prospectively assessed. The study demonstrates limited salvage activity but cannot establish neoadjuvant efficacy, MPR, pCR, or perioperative benefit.
NCT03359018, APFAO	Phase II antiangiogenic-immunotherapy combination study conducted in the advanced salvage setting.	Apatinib, a VEGFR-2 inhibitor, plus camrelizumab, a PD-1 antibody.	Forty-three patients with advanced osteosarcoma that had progressed after standard chemotherapy.	The objective response rate was 20.9%, and the 6-month progression-free survival rate was 50.9%.	Exploratory analyses suggested that selected baseline clinical or immune characteristics might be associated with progression-free survival, but these findings require validation.	The study supports potential combination activity in chemotherapy-refractory disease but does not demonstrate improved MPR, pCR, resectability, complete resection, or postoperative outcomes.
NCT07142863	Registered phase II single-arm neoadjuvant immunochemotherapy study, not yet recruiting at the time of registry verification, and designed for integration into a prospectively planned surgical pathway.	Subcutaneous envafolimab, a PD-L1 single-domain antibody, combined with protocol-defined neoadjuvant chemotherapy.	Patients with resectable stage IIB osteosarcoma.	Not yet recruiting at registry verification; no efficacy or perioperative safety results are available.	The protocol incorporates pathological response, paired assessment of the tumor immune microenvironment, and surgical feasibility into the evaluation of treatment activity.	This registered study represents a candidate true neoadjuvant design and is directly relevant to pathological response, completion of planned surgery, perioperative safety, and subsequent oncological outcomes. However, because recruitment had not begun and no results were available at the time of registry verification, no conclusions regarding efficacy or safety can currently be drawn.
NCT04897321, 3CAR	Phase I dose-escalation study of adoptive cellular immunotherapy in recurrent or refractory disease.	B7-H3-specific chimeric antigen receptor T cells.	Children and young adults aged ≤21 years with recurrent or refractory B7-H3-positive solid tumors.	Recruiting at registry verification; the study primarily evaluates dose-limiting toxicity, feasibility, and early safety and activity signals.	The trial assesses B7-H3 target feasibility, cellular-therapy pharmacodynamics, and treatment-related toxicity in a heterogeneous pediatric solid-tumor population.	The study was not designed to assess neoadjuvant pathological response, R0 resection, completion of planned surgery, or perioperative outcomes. Its relevance to osteosarcoma is mechanistic and translational rather than direct evidence of neoadjuvant benefit.
NCT04294511	Phase II single-arm study of neoadjuvant chemoimmunotherapy in a prospectively planned surgical pathway.	Camrelizumab, a PD-1 antibody, combined with neoadjuvant doxorubicin, cisplatin, ifosfamide, and methotrexate.	Planned enrollment of 75 patients with osteosarcoma.	The primary endpoint is tumor cell necrosis rate at 24 months. In the published report, 75 patients were recruited and 64 completed neoadjuvant therapy and underwent surgery, of whom 31 (48.4%) achieved a good tumor necrosis rate of at least 90%; the estimated 2-year progression-free and overall survival were 69.6% and 89.4%, and grade 3 or 4 treatment-related adverse events occurred in 62.7% (39). The registry record itself has not been updated since March 2020, still lists recruitment status as unknown, and carries no posted results.	The design uses a histological necrosis endpoint rather than the percentage of residual viable tumor, The registry record specifies no paired immune-microenvironment analysis, although the published report describes a comprehensive analysis of stromal tumor-infiltrating lymphocytes (39).	This is a true neoadjuvant design in which the investigational agent is delivered before planned resection, and it is therefore directly relevant to pathological response. However, its necrosis-based endpoint predates contemporary pan-tumor criteria (see Section 4.2), and because the trial was single-arm and non-randomized it cannot establish an incremental contribution of camrelizumab beyond intensive multiagent chemotherapy.
NCT04351308, MAPAC	Randomized phase II response-adapted escalation study conducted after induction chemotherapy in newly diagnosed disease.	MAPI plus camrelizumab versus API plus apatinib versus MAPI alone; PD-1 blockade or VEGFR-2 inhibition added on the basis of poor histological response.	Planned enrollment of 60 patients with newly diagnosed high-grade osteosarcoma showing a poor response to preoperative chemotherapy.	The primary endpoint is the 2-year event-free survival rate. The registry record was last updated in May 2020, recruitment status is listed as unknown, and no results have been posted.	Early histological response to induction chemotherapy is used as the enrichment factor, converting a routinely available prognostic readout into a patient-selection tool in the absence of a validated molecular assay.	The design is directly relevant as a template for enrichment-based neoadjuvant development and is discussed in Section 2.3. Because escalation occurs after induction chemotherapy, the investigational agent enters a microenvironment already modified by cytotoxic treatment and the remaining preoperative interval is shortened. It should be cited as a prospective framework rather than as evidence of benefit.
NCT02484443, COG AOST1421	Completed single-arm phase II study of antibody-based immunotherapy in the postsurgical recurrent-disease setting.	Dinutuximab, a GD2-directed antibody acting substantially through antibody-dependent cellular cytotoxicity, combined with granulocyte-macrophage colony-stimulating factor.	Thirty-nine eligible patients with recurrent pulmonary osteosarcoma in complete surgical remission.	The primary endpoint was the disease control rate at 12 months, compared with historical outcomes. Eleven of 39 patients remained event free, for a disease control rate of 28.2%, and the study did not demonstrate evidence of efficacy (99).	GD2 is expressed in human osteosarcoma tumors and cell lines, and the regimen depends on Fc receptor-bearing effector cells, including natural killer cells. The study therefore provides the clearest clinical test in osteosarcoma of a mechanism that requires natural killer cell function rather than HLA-mediated antigen presentation.	The study was conducted after complete surgical clearance rather than before planned resection and therefore cannot inform pathological response, MPR, pCR, or R0 resection. Its relevance is that it establishes both the feasibility and the current limits of deploying an NK-dependent mechanism at clinical scale in this disease; target expression alone was not sufficient for benefit (see Section 5.4).
NCT03013127, PROMO	Terminated phase II single-agent immunotherapy study in relapsed or metastatic disease not amenable to curative surgery.	Pembrolizumab; PD-1 pathway blockade.	Twelve patients enrolled with relapsed or metastatic osteosarcoma ineligible for curative surgery.	The primary endpoint was clinical benefit rate at 18 weeks. None of the 12 enrolled patients achieved clinical benefit, so enrollment was stopped after the first stage of a Simon two-stage design; median progression-free survival was 1.7 months and median overall survival was 6.6 months (33).	The complete absence of clinical benefit in a population selected for unresectable relapsed disease is consistent with the limited single-agent activity observed in SARC028 and reinforces that checkpoint monotherapy is insufficient in this disease. PD-L1 expression was positive in only one of 11 evaluable tumor samples, from the patient with a mixed treatment response (33).	The study cannot inform neoadjuvant pathological response because curative surgery was not feasible by design. Its inclusion is nonetheless informative for trial planning, since the pattern of early termination in unselected salvage cohorts is part of the rationale for the enrichment-based and barrier-matched designs discussed in Sections 2.3 and 5.

CAR-T, chimeric antigen receptor T cell; MPR, major pathological response; pCR, pathological complete response; PD-1, programmed cell death protein 1; PD-L1, programmed death-ligand 1; VEGFR-2, vascular endothelial growth factor receptor 2; %RVT, percentage of residual viable tumor; ADCC, antibody-dependent cellular cytotoxicity; API, doxorubicin, cisplatin, and ifosfamide; COG, Children’s Oncology Group; GD2, disialoganglioside GD2; MAPI, high-dose methotrexate, doxorubicin, cisplatin, and ifosfamide; NK, natural killer.

### 2.3 Evidence from true neoadjuvant studies

A true neoadjuvant study requires treatment to be administered before prospectively planned definitive surgery in patients with resectable disease, with surgical feasibility and pathological response explicitly assessed and reported. According to this definition, the evidence base for neoadjuvant immunotherapy in osteosarcoma remains limited, but it is no longer completely confined to theoretical extrapolation. Many studies commonly cited in discussions of preoperative immunotherapy were conducted in advanced-disease or salvage settings and therefore should not be interpreted as direct evidence supporting a neoadjuvant strategy.

The most informative published true neoadjuvant dataset is the prospective, single-arm, exploratory phase 2 trial NCT04294511, which evaluated camrelizumab combined with doxorubicin or liposomal doxorubicin, cisplatin, high-dose methotrexate, and ifosfamide plus mesna in patients with resectable osteosarcoma (39). This study is important because immunotherapy was embedded within a predefined perioperative pathway rather than introduced after failure of standard therapy. Eligible patients received neoadjuvant chemoimmunotherapy, followed by surgery 12–14 days after completion of preoperative treatment, with adjuvant therapy initiated 2–3 weeks postoperatively (39). Thus, the trial directly addressed two questions central to the neoadjuvant setting: whether PD-1 blockade can be combined with intensive osteosarcoma chemotherapy without compromising the surgical window, and whether this combination can improve pathological response in the resection specimen.

A total of 75 patients were recruited, and 64 patients completed neoadjuvant therapy and underwent surgery. Among these 64 patients, 31 achieved a good tumor necrosis rate, defined as TNR ≥90%, corresponding to a pathological response rate of 48.4% (39). With a median follow-up of 22.4 months, the estimated 2-year progression-free survival and overall survival rates were 69.6% and 89.4%, respectively (39). grade 3 or 4 treatment-related adverse events occurred in 62.7% of patients and were mainly hematologic, including decreased platelet count and decreased white blood cell count. No immune-related serious adverse events were observed (39). These findings suggest that camrelizumab-based chemoimmunotherapy is feasible and tolerable within a curative-intent surgical pathway.

Nevertheless, this study should be interpreted cautiously. Because it was a non-randomized single-arm trial, it cannot determine whether camrelizumab provided an incremental benefit beyond intensive multiagent chemotherapy. Moreover, the authors noted that this combined therapeutic strategy may not increase the tumor necrosis rate, and the potential long-term survival benefit requires further follow-up. The encouraging estimated 2-year overall survival rate should therefore be regarded as an early survival signal rather than definitive evidence of survival benefit. In addition, pathological response was assessed using the traditional osteosarcoma necrosis threshold rather than immune-adapted measures such as continuous percentage of residual viable tumor, major pathological response (MPR), or pathological complete response (pCR). Therefore, NCT04294511 provides important feasibility data and early clinical evidence for neoadjuvant chemoimmunotherapy in resectable osteosarcoma, but it does not yet establish PD-1 blockade as a new standard component of neoadjuvant treatment.

The registered phase 2 trial NCT07142863 extends this line of investigation by evaluating subcutaneous envafolimab, a single-domain PD-L1 antibody-Fc fusion protein, combined with NACT in 23 patients with resectable stage IIB osteosarcoma. The study is relevant because the investigational agent is embedded within a prospectively planned surgical pathway, with pathological response as the primary endpoint and paired assessment of the TIME and surgical feasibility among the secondary assessments.

Its route of administration raises a specific design question rather than representing a purely logistical convenience. Phase 1 data indicate that subcutaneous envafolimab is absorbed slowly, with median times to maximum serum concentration of approximately 4–7 days across dose groups and median terminal half-lives after a single dose ranging from approximately 13–29 days, and that exposure increases dose-proportionally over the studied range (40). That trial also evaluated a fixed 300 mg dose administered every 4 weeks alongside weight-based regimens (40). These properties permit fixed dosing at 2-week or 4-week intervals and avoid repeated intravenous infusions during a chemotherapy schedule that already requires prolonged inpatient hydration and reliable central venous access. In non-small cell lung cancer, randomized comparisons of subcutaneous and intravenous PD-1 or PD-L1 blockade have demonstrated noninferior pharmacokinetic exposure with comparable efficacy and safety, supporting the general feasibility of route substitution (41, 42).

The same pharmacokinetic profile, however, carries perioperative disadvantages that warrant explicit protocol planning. Slow absorption combined with a prolonged terminal half-life means that meaningful drug exposure persists for several weeks after the final preoperative dose. The agent therefore cannot be rapidly withdrawn if an immune-related adverse event emerges or if surgery must be brought forward, a consideration that is more consequential in a curative-intent surgical pathway than in the advanced-disease setting in which these agents were developed. Within a preoperative window of approximately 12 weeks, delayed attainment of steady state may also concentrate effective immunological exposure within the later treatment cycles, which complicates attribution of any pathological effect to a defined exposure period. Subcutaneous bioavailability may additionally vary with injection site, subcutaneous tissue mass, and nutritional status, all of which are relevant in adolescents receiving intensive MAP chemotherapy, while injection-site reactions and anti-drug antibody formation are route-specific concerns. Neoadjuvant trials of subcutaneous checkpoint blockade should therefore prespecify the interval between the last dose and surgery, and should report perioperative drug exposure alongside pathological response rather than assuming equivalence with intravenous scheduling.

It is also notable that NCT07142863 defines its primary endpoint using the conventional Huvos-based necrosis threshold rather than continuous %RVT, which illustrates how prevailing osteosarcoma practice still diverges from contemporary pan-tumor pathological assessment (see Section 4.2). Because the study was not yet recruiting at the time of registry verification, no conclusions regarding efficacy, pathological response, or surgical safety can currently be drawn.

A conceptually different strategy is the response-adapted escalation design represented by NCT04351308. This randomized phase 2 trial enrolls patients with newly diagnosed high-grade osteosarcoma who show a poor response to initial preoperative chemotherapy, and compares MAPI (high-dose methotrexate, doxorubicin, cisplatin, and ifosfamide) plus camrelizumab, API (doxorubicin, cisplatin, and ifosfamide) plus apatinib, and MAPI alone, with 2-year event-free survival as the primary endpoint.

The design merits attention because it addresses a problem that biomarker science has not yet solved. No validated molecular assay currently identifies, before treatment begins, which patients with osteosarcoma will fail to respond to MAP chemotherapy. Early histological and radiological response to induction chemotherapy is, by contrast, an inexpensive, universally available, and prognostically established readout. Using it as an enrichment factor converts a measurement that is already made in routine care into a patient-selection tool, and thereby concentrates investigational exposure on the subgroup with the greatest unmet need.

This approach offers both scientific and ethical advantages over adding an ICI to every patient. Scientifically, restricting randomization to poor responders raises the expected event rate and therefore the statistical efficiency of a trial conducted in a rare disease, and it avoids diluting a treatment effect with patients who would have done well on chemotherapy alone. It also yields an internally interpretable comparison, because all randomized patients have demonstrated the same chemotherapy-refractory phenotype. Ethically, it avoids exposing chemosensitive patients, whose prognosis under standard care is already relatively favorable, to the additional toxicity, cost, and risk of surgical delay associated with immune checkpoint blockade for no anticipated gain. Because intensifying conventional cytotoxic therapy in poor responders has not improved outcome (9), testing a mechanistically non-cross-resistant intervention in precisely that population is the logical successor question. We therefore regard response-adapted escalation as a template for future osteosarcoma immunotherapy trials rather than as merely one ongoing study, and we suggest that subsequent perioperative protocols in this disease adopt an equivalent enrichment step in preference to unselected addition of an ICI.

Two limitations temper this recommendation. Escalation occurs only after induction chemotherapy, so the investigational agent is delivered into a microenvironment already modified by cytotoxic treatment, and the preoperative interval remaining for immune activation is correspondingly shortened; a response-adapted design therefore tests immunotherapy under less favorable immunological conditions than a *de novo* neoadjuvant design would. In addition, the registry record for NCT04351308 has not been updated since May 2020, its recruitment status remains listed as unknown, and no results have been reported. The design should accordingly be cited as a prospective framework worth emulating rather than as evidence of benefit.

Overall, the current true neoadjuvant evidence suggests that chemoimmunotherapy is feasible in carefully selected patients with resectable osteosarcoma, but it does not yet establish a new standard of care. Future studies should prospectively define the duration of preoperative treatment, the maximum acceptable delay to surgery, criteria for early treatment discontinuation, standardized specimen-processing methods, and thresholds for major pathological response and pathological complete response. Pathological findings should be correlated with R0 resection rates, postoperative morbidity, circulating tumor DNA dynamics, event-free survival, and overall survival. Such a design is necessary to determine whether an early biological response translates into durable clinical benefit rather than merely radiological or histological changes.

Equally important, a neoadjuvant immunotherapy program must be judged against the objectives that preoperative treatment already achieves rather than against immunological endpoints alone. A regimen that enhances immune activation but reduces the proportion of patients who complete successful limb-salvage surgery, or that permits micrometastatic progression during a prolonged preoperative interval, would represent a net clinical loss irrespective of its immunological effect. Pathological response, limb-salvage rate, time to surgery, and metastasis-free survival should therefore be reported together and interpreted jointly, so that gains in one domain are not obtained at an unrecognized cost in another.

### 2.4 Clinical scope in previously treated and metastatic osteosarcoma

The clinical meaning of the term “neoadjuvant therapy” requires particular clarification in previously treated patients. In treatment-naive patients with localized, resectable osteosarcoma, neoadjuvant therapy refers to systemic treatment administered before the prospectively planned definitive resection of the primary tumor. By contrast, patients with local recurrence or metachronous metastatic disease have generally already undergone definitive resection of the primary tumor and systemic chemotherapy. Therefore, when an investigational immunotherapy regimen is administered before the resection of recurrent or metastatic lesions, the terms “preoperative salvage therapy” or “perioperative immunotherapy” may be more precise than “neoadjuvant therapy” in its conventional sense.

Most clinical evidence regarding ICIs in osteosarcoma has been derived from treatment-experienced patients with advanced, recurrent, or chemotherapy-refractory disease. SARC028, KEYNOTE-051, the phase 2 pembrolizumab study conducted by Boye et al., and the APFAO trial primarily evaluated immunotherapy as salvage systemic treatment rather than as a component of a prospectively planned surgical pathway (33, 34, 36, 38). These studies demonstrate the feasibility of administering immune checkpoint blockade or immunotherapy-based combinations to previously treated patients. However, the generally low response rates do not establish that these treatments can reliably downstage disease, increase complete resection rates, or improve postoperative outcomes.

Nevertheless, preoperative immunotherapy may be investigated in carefully selected, treatment-experienced patients with technically resectable local recurrence or limited metastatic disease. Potential candidates should have an adequate performance status and sufficient organ reserve, a realistic prospect of complete surgical resection, and a preoperative treatment interval that does not compromise curative-intent surgery. Previous treatment may alter immune cell infiltration and immune checkpoint expression (19). However, it may also select for resistant clones, increase temporal and spatial heterogeneity, and create molecular or immunological discordance between archival primary tumor tissue and current recurrent or metastatic lesions. Therefore, whenever feasible, biomarker assessment should be performed using a newly obtained specimen from the lesion targeted for treatment.

Treatment-experienced patients also present distinct safety and surgical challenges. Cumulative treatment-related toxicities may compromise bone marrow, cardiac, renal, hepatic, or pulmonary reserve. In addition, combinations of ICIs, targeted agents, and salvage chemotherapy may result in overlapping toxicities. Immune-related adverse events or pseudoprogression may delay potentially curative surgery and complicate the assessment of tumor resectability. Therefore, clinical protocols should specify a predefined maximum interval before surgery, early response reassessment, multidisciplinary review, and explicit criteria for discontinuing investigational therapy and proceeding directly to surgical resection (43–45).

Perioperative immunotherapy may also be investigated in selected patients with metastatic osteosarcoma. However, its feasibility depends primarily on whether all known sites of disease remain amenable to complete local control. Patients with limited synchronous pulmonary metastases may remain eligible for curative-intent multimodal therapy when complete resection of both the primary tumor and all metastatic deposits is technically feasible. The established use of preoperative chemotherapy followed by surgery in patients with metastatic osteosarcoma at diagnosis supports the general feasibility of a preoperative systemic treatment framework (11). Nevertheless, current evidence does not establish that the addition of immunotherapy improves resectability or survival.

Evidence from relapsed osteosarcoma indicates that achieving a second complete surgical remission is a major determinant of survival after relapse. In an analysis by the Cooperative Osteosarcoma Study Group, patients who achieved a second complete surgical remission had substantially better survival than those who did not. A longer interval to relapse and a lower tumor burden were also associated with a more favorable prognosis (46). In patients with pulmonary metastatic disease, complete metastasectomy, fewer pulmonary lesions, and a longer disease-free interval were associated with improved survival. Repeated pulmonary metastasectomy may also provide long-term survival and the possibility of cure in appropriately selected children, adolescents, and young adults (47).

Accordingly, in resectable recurrent or oligometastatic disease, immunotherapy should be evaluated as an experimental adjunct to complete local control rather than as a substitute for it. In patients with diffuse, rapidly progressive, or technically unresectable metastatic disease for whom no definitive surgical procedure is planned, immunotherapy should be classified as salvage or palliative systemic treatment. Relevant endpoints in this setting include objective response, disease control, progression-free survival, overall survival, symptom control, and quality of life, rather than pathological response or surgical margin status.

Future trials should avoid combining treatment-naive localized disease, previously treated local recurrence, resectable oligometastatic disease, and unresectable disseminated disease within a single undifferentiated cohort. Stratification factors should include previous systemic treatment regimens, response to chemotherapy, disease-free interval, timing and burden of metastatic disease, baseline resectability, and the feasibility of complete surgical clearance. Perioperative studies should additionally report the completion rate of planned surgery, time to surgery, rates of R0 resection or complete metastasectomy, treatment-related surgical delays, postoperative complications, lesion-specific pathological responses, and circulating tumor DNA dynamics.

## 3 Barriers to clinically meaningful pathological response

The limited therapeutic activity observed in current studies appears to reflect a series of interconnected barriers rather than a single dominant mechanism. Achieving a clinically meaningful response requires immunologically recognizable antigens, functional antigen presentation, the recruitment and activation of tumor-reactive effector cells, reversal of local immunosuppression, safe completion of definitive surgery, and accurate assessment of treatment response. Failure at any stage of this process may prevent biological activity from translating into deep pathological regression.

### 3.1 Antigenicity, HLA presentation, and biomarker uncertainty

At the population level, osteosarcoma generally exhibits a relatively low tumor mutational burden (TMB), although substantial intertumoral variability exists and a subset of tumors may exhibit comparatively higher values (24, 25). TMB quantifies the burden of somatic mutations but does not indicate whether mutation-derived antigens are expressed, processed, presented by HLA molecules, or recognized by tumor-reactive T cells. Similarly, the extensive copy-number alterations and structural rearrangements characteristic of osteosarcoma do not consistently generate neoantigens that are detectably expressed and immunologically recognizable (24).

Defective antigen presentation represents an additional and partly independent barrier to effective antitumor immunity. Osteosarcoma cells may downregulate or lose HLA class I expression, thereby impairing their recognition and elimination by tumor-specific CD8+ T cells. Reduced HLA class I expression has been observed in both primary and metastatic osteosarcoma and has been associated with unfavorable clinical outcomes (23). Therefore, resistance to immune checkpoint blockade cannot be attributed solely to low TMB. Instead, it likely reflects the combined effects of heterogeneous antigen expression, defective HLA-mediated antigen presentation, quantitatively or functionally inadequate T-cell infiltration, and myeloid-mediated immunosuppression (19, 27, 28).

These biological limitations also explain why neither TMB nor PD-L1 is sufficient as a standalone biomarker for patient selection. In a small sequencing study, a cohort-specific high-TMB category was associated with survival. However, that study did not evaluate response to immunotherapy, and its cohort of 31 patients was neither powered nor stratified to separate the contribution of tumor mutational burden from established clinicopathological risk factors (25). PD-L1 expression varies across patients and specimens, as well as between tumor cells and infiltrating immune cells, and may change dynamically in response to inflammatory signaling or systemic treatment (19, 24, 35, 48). PD-L1 positivity does not ensure the presence of functional tumor-reactive T cells, whereas a negative result from a limited biopsy does not exclude immune checkpoint-mediated suppression.

Biomarker development should therefore shift toward an integrated model that reflects the multistep nature of antitumor immunity. Candidate components include the burden of detectably expressed antigens, HLA class I expression, the integrity of antigen-processing pathways, interferon-γ-related or T-cell-inflamed gene signatures, T-cell receptor clonality and specificity, and the density, functional state, and spatial organization of immune cells (23–25, 48, 49). Paired pretreatment biopsy and post-treatment resection specimens provide an opportunity to evaluate these features dynamically rather than relying solely on a single baseline sample.

Tertiary lymphoid structures (TLS) may provide complementary information by reflecting organized local interactions among B cells, T cells, dendritic cells, plasma cells, and stromal components (50–52). However, the strongest evidence linking TLS to immunotherapy response in sarcoma has been derived from soft-tissue sarcoma rather than osteosarcoma. A transcriptomic analysis identified a B-cell-rich sarcoma immune class characterized by TLS, which was associated with improved survival and a favorable response to pembrolizumab (53). Subsequently, a prospectively TLS-selected cohort in the PEMBROSARC study achieved a 6-month non-progression rate of 40% and an objective response rate of 30%, compared with 4.9% and 2.4%, respectively, in previously reported unselected cohorts (54).

These findings support the prospective evaluation of TLS presence, maturation status, and cellular composition in osteosarcoma. However, the results should not be directly extrapolated across different sarcoma subtypes. TLS should therefore be considered a candidate component of a composite biomarker framework rather than a substitute for the assessment of HLA competence, antigen expression, T-cell functionality, or other dimensions of immune competence.

### 3.2 Myeloid, metabolic, and stromal immunosuppression

The osteosarcoma TIME is frequently characterized by a predominance of myeloid cell populations. M2-polarized tumor-associated macrophages may constitute a substantial proportion of the infiltrating immune cell population and suppress cytotoxic lymphocyte activity through IL-10, transforming growth factor β (TGF-β), immune checkpoint ligands, and other inhibitory mediators (28–30). Myeloid-derived suppressor cells and regulatory T cells further reinforce this immunosuppressive state, forming a complex network that is unlikely to be reversed by PD-1/PD-L1 blockade alone.

The stromal compartment adds a further layer of immunosuppression. In osteosarcoma, tumor-associated macrophages promote angiogenesis and tumor invasion through vascular endothelial growth factor (VEGF) signaling and the secretion of matrix-remodeling enzymes (28, 29), linking immunosuppression to vascular dysfunction and metastatic progression.

Metabolic abnormalities within the TIME further impair immune effector function. Rapid tumor growth and abnormal vasculature contribute to hypoxia, extracellular acidosis, nutrient competition, and the accumulation of extracellular adenosine. Adenosinergic signaling is itself a recognized mechanism of immunosuppression within the tumor microenvironment: CD73 is expressed on immunosuppressive populations including regulatory T cells and myeloid-derived suppressor cells, and intratumoral CD73 expression is often associated with a poor clinical prognosis (31). Collectively, these myeloid, metabolic, and stromal barriers help explain why the presence of immune-cell infiltration does not necessarily translate into effective tumor eradication.

### 3.3 Genomic instability and immune recognition

Osteosarcoma is defined less by recurrent point mutations than by structural disruption of the genome. Whole-genome sequencing shows a landscape dominated by complex structural variation and copy-number alteration rather than by a small set of recurrent driver substitutions, with localized hypermutation in half of tumors and near-universal disruption of the p53 pathway, frequently through translocation within the first intron of TP53 (55). Chromothripsis-associated rearrangement and amplification recur at discrete genomic regions and themselves generate driver mutations (56). This architecture determines both the raw material available to the immune system and several of the mechanisms that conceal it.

The immunological consequences are bidirectional, which is why genomic instability cannot be treated as a simple predictor of benefit. Chromosomal instability generates micronuclei whose rupture releases genomic DNA into the cytosol and activates cyclic GMP-AMP synthase (cGAS)–stimulator of interferon genes (STING) signaling; in the same study, however, continuous segregation errors promoted invasion and metastasis in a STING-dependent manner rather than immune clearance (57). Extracellularly, ENPP1 hydrolyzes immunostimulatory cGAMP into products including adenosine, and its loss restored immune infiltration and potentiated checkpoint blockade whereas its overexpression conferred resistance (58). Chronic activation of an innate pathway may therefore be immunosuppressive rather than immunogenic, so an interferon signature in a pretreatment specimen is not by itself a favorable finding.

Aneuploidy constrains patient selection. In an analysis of 12 human cancer types, highly aneuploid tumors showed reduced expression of markers of cytotoxic infiltrating immune cells in most of those types, particularly CD8+ T-cell markers, and aneuploidy correlated with reduced response to immunotherapy (59). Osteosarcoma-specific profiling is consistent: immune infiltrate and T-cell receptor clonality were low, expressed neoantigen was lacking and significantly associated with nonsense-mediated decay, and samples with low infiltrate carried more deleted genes (24). Allele-specific HLA loss of heterozygosity, reported in 40% of non-small cell lung cancers (60), indicates further that antigen supply and antigen presentation can fail independently. Mutational and structural-variation burden should therefore not be treated as interchangeable surrogates for immunogenicity.

### 3.4 Surgical and radiological constraints

Neoadjuvant immunotherapy is administered within a curative-intent treatment window in which failure to complete definitive surgery may outweigh any uncertain systemic benefit. Immune-related adverse events, including dermatitis, colitis, hepatitis, pneumonitis, and endocrine dysfunction, may necessitate treatment interruption or immunosuppressive therapy. In a prospective neoadjuvant trial of nivolumab in resectable head and neck squamous cell carcinoma, no patient experienced a protocol-defined treatment-related surgical delay beyond four weeks (61). That reassurance should not be extrapolated uncritically to osteosarcoma, in which the preoperative window is longer, chemotherapy is given concurrently, and reconstruction is more complex. Combination regimens may further increase both the incidence and clinical complexity of these adverse events.

Treatment-induced local inflammation and fibrosis may also distort normal tissue planes. Dense adhesions between the tumor and adjacent muscles, fascia, or neurovascular structures may increase operative complexity and compromise the feasibility of achieving the intended surgical margin or performing limb-salvage surgery. Studies in other solid tumors suggest that neoadjuvant immunotherapy may affect operative time, intraoperative blood loss, wound complications, and postoperative morbidity (43, 44). Osteosarcoma surgery, however, raises reconstruction-specific questions that cannot be resolved by extrapolation from nonskeletal tumors.

Almost all patients undergoing limb salvage receive a metallic endoprosthesis, a massive allograft, or an allograft-prosthesis composite, and the durability of each depends on biological integration at the host bone interface. Whether checkpoint blockade alters this process is unknown, and in our view it is the most important unresolved surgical question in this field. Two considerations make it non-trivial. First, immune activation and the pro-inflammatory cytokine milieu it generates act on the same osteoclast-osteoblast signaling axis that governs allograft creeping substitution and peri-implant osseointegration, so an effect in either direction is biologically plausible and cannot be assumed to be neutral. Second, the corticosteroids and other immunosuppressive agents used to treat immune-related adverse events are themselves associated with impaired wound healing and increased infection risk, and their administration may cluster precisely within the perioperative period. These concerns are amplified by the high baseline complication burden of pediatric limb salvage: in a recent series of 84 children followed for a median of 46 months, the all-cause complication rate was 75%, the major complication rate was 36%, and the reoperation rate was 55%, with 71% of patients sustaining a first complication within 1 year of surgery (62). Any incremental risk attributable to immunotherapy would therefore be superimposed on an already high event rate and could be detected only through prespecified, standardized reporting of reconstruction-specific outcomes, including deep periprosthetic infection, aseptic loosening, allograft nonunion or fracture, and time to unrestricted weight-bearing.

The preoperative interval itself carries a second and distinctly orthopedic risk. Osteosarcoma structurally weakens the cortical bone it occupies, and pathological fracture occurs in a clinically meaningful minority of patients. In a meta-analysis of 7,604 patients with osteosarcoma, 12% had a pathological fracture, and fracture was associated with lower 3-year and 5-year overall survival, with relative risks of 1.53 and 1.27, respectively, and with an increased risk of metastasis, with a relative risk of 1.33; limb-salvage surgery was nevertheless not associated with a higher rate of local recurrence in appropriately selected patients (63). Any extension of the preoperative window, whether to deliver additional immunotherapy cycles, to allow an immune-related adverse event to resolve, or to repeat equivocal imaging, prolongs the period during which a structurally compromised bone continues to bear load. Neoadjuvant immunotherapy protocols in osteosarcoma should therefore include fracture-risk assessment and explicit weight-bearing instructions at enrollment, and should report pathological fracture occurring during the preoperative interval as a protocol-defined safety event rather than as an incidental surgical complication. All of these risks require prospective evaluation within orthopedic oncology and should not be inferred from experience with nonskeletal tumors.

Radiological interpretation presents a parallel challenge. Immune cell infiltration, inflammatory edema, reparative fibrosis, or an osteoblastic flare may increase lesion size, density, or radionuclide uptake without indicating progression of viable tumor. Conversely, stable lesion dimensions may conceal persistent viable tumor within a mineralized matrix. Conventional measurements based on the Response Evaluation Criteria in Solid Tumors (RECIST) may therefore be insufficient to determine whether treatment should be continued or surgery should proceed (45). Pooled data across solid tumors offer partial quantitative guidance: pseudoprogression during checkpoint blockade typically occurs early, at a pooled median of 2.52 months, is accompanied by a mean increase in tumor burden of approximately 33%, and only rarely exceeds a doubling of tumor burden (64). In a patient with resectable osteosarcoma, marked or late radiological growth should therefore be treated as probable true progression and should prompt surgery rather than continued observation.

Metabolic imaging offers a more specific readout than anatomic measurement in this setting, because it reports cellular activity rather than the dimensions of a mineralized mass. In extremity osteosarcoma treated with conventional NACT, the maximum standardized uptake value measured after chemotherapy, conventionally denoted SUV**_2_** (the pretreatment value being denoted SUV**_1_**), correlates with histological response: an SUV**_2_** above 5 identified the majority of histological nonresponders with a positive predictive value of 88.9%, and necrosis at the point of maximum metabolic activity correlated with the histological response of the entire resected specimen (65). A prospective model combining ^18^F-FDG PET with MRI further indicated that an SUV**_2_** of 2 or below predicted a good histological response and an SUV**_2_** above 5 predicted a poor response, with the intermediate group resolved using the metabolic volume change ratio (66). Two caveats limit direct transfer of these thresholds to immune-based regimens. Absolute SUV cut-offs are scanner- and protocol-dependent, so ratio metrics such as SUV**_2_**/SUV**_1_** or the percentage change from baseline are more transferable between institutions. The second caveat is more consequential: these thresholds were derived in cytotoxically treated tumors, whereas immune activation increases glucose uptake by infiltrating lymphocytes and macrophages and can therefore raise SUV**_max_** independently of viable tumor burden. Serial PET performed at defined timepoints, interpreted according to the direction and magnitude of change rather than a single absolute value, is consequently the more informative approach, and PET-based thresholds require prospective recalibration in osteosarcoma treated with immunotherapy.

Liquid biopsy provides an orthogonal, tumor-derived signal that is not confounded by inflammation. Tumor-informed deep sequencing of circulating tumor DNA (ctDNA) detects minimal residual disease and predicts relapse in osteosarcoma (67), and serial measurement has been shown to track treatment-related change longitudinally in prospective pediatric sarcoma cohorts (68). In principle, ctDNA is well suited to the preoperative window: a molecular response preceding or exceeding the radiological response would support continuing treatment through an equivocal scan, whereas rising ctDNA during preoperative therapy would argue for proceeding directly to surgery. The current evidence does not yet support this application. Available osteosarcoma data establish the prognostic value of postoperative ctDNA status rather than the preoperative prediction of pathological response, and in a prospective pediatric sarcoma cohort a single end-of-treatment measurement did not correlate with radiological or clinical response (68). Preoperative prediction of MPR or pCR from ctDNA therefore remains an explicit research objective rather than an established use, and its evaluation requires serial sampling anchored to defined treatment timepoints and paired directly with %RVT in the resection specimen. Assay design is a further constraint, because osteosarcoma is driven predominantly by structural and copy-number alterations rather than recurrent point mutations, which favors tumor-informed panels over fixed hotspot assays.

No single modality resolves this uncertainty. Diffusion-weighted magnetic resonance imaging, metabolic imaging, serial anatomic imaging, and ctDNA analysis provide complementary and partly independent information on tumor cellularity, metabolic activity, and molecular disease burden (65–67, 69), and disagreement among them is itself informative: concordant metabolic and molecular response supports continuing preoperative treatment, whereas discordance should trigger multidisciplinary review rather than further observation. Clinical protocols should nevertheless specify a maximum duration of preoperative treatment and predefined criteria for surgical reassessment, so that ambiguous imaging findings do not result in the loss of an opportunity for curative surgery.

These barriers affect not only whether a tumor responds but also how that response should be assessed. Radiological changes may be confounded by inflammatory or osteoblastic remodeling, whereas conventional necrosis-based grading may not adequately distinguish residual viable tumor from an immune-mediated tumor regression bed. Therefore, a coherent neoadjuvant framework requires a pathology-centered yet multidimensional definition of treatment success.

## 4 Pathological response as a central but unvalidated early endpoint

A defining advantage of the neoadjuvant setting is that the resected tumor provides a direct biological measure of treatment activity. Pathological response should therefore be a central endpoint in neoadjuvant immunotherapy trials, not merely a secondary descriptive measure. Nevertheless, pathological response should not be regarded as the sole therapeutic objective. The ultimate clinical goals remain complete oncological resection, successful completion of the planned surgical procedure, control of micrometastatic disease, and improvements in event-free survival and overall survival.

### 4.1 Limitations of conventional necrosis-based assessment

In osteosarcoma treated with conventional NACT, histological response is assessed primarily according to the proportion of tumor necrosis, with a necrosis rate of at least 90% generally considered indicative of a favorable response (6, 70, 71). Although this approach has established prognostic value in chemotherapy-treated osteosarcoma, it may be insufficient for evaluating immune-based regimens.

Immune checkpoint blockade and other immunomodulatory therapies may induce lymphocytic and macrophage infiltration, fibroinflammatory remodeling, cholesterol cleft formation, granulation tissue, neovascularization, and other features of a treatment-associated regression bed. Therefore, exclusive reliance on coagulative necrosis may not adequately distinguish residual viable tumor from immune-mediated tissue remodeling. Conversely, dense inflammatory infiltrates or reparative tissue may be misclassified as persistent viable malignancy.

Pathological assessment is further complicated by the mineralized osteoid matrix and pronounced spatial heterogeneity of osteosarcoma. Viable tumor cells may persist within regions that appear necrotic, fibrotic, or calcified, while different areas of the same specimen may exhibit markedly heterogeneous treatment effects. Standardized specimen mapping and comprehensive tissue sampling are therefore essential to minimize the risk of response misclassification.

### 4.2 Integrating %RVT, MPR, and pCR

The International Association for the Study of Lung Cancer (IASLC) framework for lung cancer, an earlier organ-specific recommendation, standardized assessment of the percentages of viable tumor, necrosis, and stroma, together summing to 100% (72). Building on those principles, the 2026 Society for Immunotherapy of Cancer (SITC) and International Neoadjuvant Melanoma Consortium (INMC) pan-tumor framework quantifies pathological response as %RVT, necrosis, and regression, extending these recommendations across tumor types after neoadjuvant therapy (73). In many neoadjuvant studies, MPR is operationalized as 10% RVT or less, whereas pCR is defined as 0% RVT (72, 73). However, these thresholds have not been prospectively validated in osteosarcoma treated with neoadjuvant immunotherapy. This is why continuous %RVT should be reported. Binary classification alone may obscure clinically meaningful differences among near-complete, partial, and minimal responses. The key differences between conventional necrosis-based grading and the pan-tumor pathological response assessment framework are summarized in **Table 2**.

**Table 2:** Comparison of conventional osteosarcoma histological grading and the potential application of pan-tumor pathological response assessment criteria in neoadjuvant immunotherapy.

Assessment dimension	Conventional necrosis-based grading in osteosarcoma, including the Huvos system and related response classifications	2026 SITC/INMC pan-tumor pathological response assessment framework	Clinical and translational significance for neoadjuvant immunotherapy in osteosarcoma
Core assessment object	Primarily evaluates the proportion of treatment-induced tumor necrosis.	Comprehensively evaluates the percentage of residual viable tumor (%RVT), necrosis, and the treatment-associated regression bed.	Conventional necrosis-based grading may not fully capture immune-cell infiltration, fibroinflammatory remodeling, or other immune-related treatment effects. The pan-tumor framework may help distinguish residual viable tumor from necrosis and regression-bed changes.
Candidate early-response thresholds	A favorable histological response is conventionally defined as a tumor necrosis rate of ≥90%.	Major pathological response (MPR) is commonly defined as ≤10% RVT, whereas pathological complete response (pCR) is defined as 0% RVT. Continuous %RVT should also be reported.	Continuous %RVT, MPR, and pCR provide standardized candidate early-response measures. However, none has been prospectively validated as a surrogate for event-free survival or overall survival in osteosarcoma treated with neoadjuvant immunotherapy.
Histopathological features evaluated	Focuses mainly on cytotoxic injury, including coagulative necrosis, nuclear fragmentation, membrane disruption, and loss of tissue architecture.	Evaluates residual viable tumor together with foamy macrophages, lymphoplasmacytic infiltration, cholesterol clefts, fibrosis, granulation tissue, neovascularization, and other components of the treatment-associated regression bed.	A multidimensional assessment may better distinguish direct cytotoxic necrosis from immune-mediated and stromal remodeling. However, immune-cell infiltration should not be assumed to indicate tumor-specific immunity.
Specimen sampling and mapping	Uses representative sections across the tumor, with the sampling strategy adapted to tumor size and anatomical complexity.	The pan-tumor framework recommends complete histological submission of tumors ≤3 cm and extensive standardized mapping and sampling of larger tumors.	Standardized mapping is particularly important in osteosarcoma because mineralized osteoid, fibrosis, necrosis, and viable tumor may be distributed heterogeneously throughout the resection specimen.
Role in clinical-trial design	Provides an established prognostic assessment after conventional neoadjuvant chemotherapy.	Provides a candidate framework for assessing early pathological activity after neoadjuvant immune-based treatment.	Pathological response should be interpreted together with completion and timing of planned surgery, R0 resection, perioperative morbidity, ctDNA dynamics or clearance, event-free survival, overall survival, and functional outcomes.
Current validation status in osteosarcoma	The prognostic relevance of chemotherapy-induced necrosis has been established in conventional osteosarcoma treatment.	The framework is derived from pan-tumor recommendations and experience in other malignancies.	Its feasibility, reproducibility, optimal sampling requirements, response thresholds, and association with long-term outcomes require prospective validation in osteosarcoma-specific neoadjuvant immunotherapy trials.

ctDNA, circulating tumor DNA; INMC, International Neoadjuvant Melanoma Consortium; MPR, major pathological response; pCR, pathological complete response; SITC, Society for Immunotherapy of Cancer; %RVT, percentage of residual viable tumor.

A terminological clarification is required before these frameworks can be applied consistently in osteosarcoma. This literature has historically reported treatment effect as the tumor necrosis rate (TNR), the proportion of the specimen showing treatment-induced necrosis, with a value of at least 90% denoting a favorable response (8, 9). Contemporary pan-tumor frameworks instead report %RVT, which quantifies what remains rather than what has been destroyed (72, 73). The two measures are not simple complements, because the non-viable fraction of a treated specimen comprises necrosis together with fibrosis, granulation tissue, foamy macrophages, cholesterol clefts, and lymphoplasmacytic infiltrate, components that necrosis-based scoring does not separately resolve and that are precisely the features expected to increase after immune-based treatment. %RVT is accordingly the preferred term within modern pathological assessment frameworks, whereas TNR should be regarded as a historical, disease-specific term. We adopt %RVT throughout the remainder of this review and retain TNR only when reporting studies that originally used it. We further recommend that future osteosarcoma trials report continuous %RVT as the primary pathological measure, while retaining the 90% necrosis category as a secondary descriptor solely to preserve comparability with historical cohorts.

Studies in melanoma and mismatch repair-deficient colorectal cancer suggest that MPR and pCR following neoadjuvant immunotherapy are associated with favorable long-term outcomes (13, 14, 74). However, these findings cannot be directly extrapolated to osteosarcoma. Neither MPR nor pCR has been prospectively validated as a surrogate endpoint for event-free survival or overall survival in patients with osteosarcoma treated with neoadjuvant immunotherapy. Accordingly, pCR should be regarded as a potentially valuable early efficacy signal rather than a validated substitute for long-term clinical benefit.

The interpretation of pCR should be separated from the concept of durability. pCR reflects the absence of viable tumor in the resection specimen at a defined time point, whereas durable disease control can only be determined through longitudinal follow-up. Thus, pCR should be regarded as an early pathological endpoint rather than a surrogate for cure. Pathological findings should be interpreted in conjunction with event-free survival, overall survival, recurrence patterns, and molecular indicators of residual disease.

### 4.3 A multidimensional definition of neoadjuvant success

Future osteosarcoma trials should not define successful neoadjuvant treatment solely on the basis of pCR. A clinically meaningful endpoint framework should instead integrate four complementary domains.

First, pathological efficacy should be assessed using continuous %RVT, MPR, pCR, the extent and cellular composition of the treatment-associated regression bed, and the spatial heterogeneity of response across the resection specimen. Second, surgical feasibility and safety should be evaluated according to completion of the planned operation, time to surgery, achievement of R0 resection, preservation of the intended limb-salvage procedure, operative complexity, wound complications, reconstruction-related morbidity, and treatment-related surgical delays.

Third, molecular and immunological responses should encompass circulating tumor DNA clearance, restoration of antigen-presentation pathways, expansion of tumor-reactive rather than bystander T-cell clones, and spatial reorganization of the immune and stromal compartments. Fourth, long-term clinical benefit should be assessed using event-free survival, metastasis-free survival, overall survival, local control, and functional outcomes. These endpoints are essential for determining whether an early pathological response translates into durable clinical benefit.

This multidimensional framework clarifies the role of pCR within the broader argument of this review. pCR is not the biological purpose of immunotherapy and should not be presented as the sole therapeutic objective. Rather, it represents the most stringent category within a continuum of pathological response and should be interpreted in conjunction with surgical outcomes, molecular clearance, treatment-related toxicity, and long-term survival. This framework provides a common basis for determining whether future therapeutic strategies overcome specific biological or clinical barriers and produce concordant improvements across pathological, molecular, surgical, and long-term clinical endpoints.

## 5 Barrier-matched strategies for next-generation trials

The diversity of resistance mechanisms argues against evaluating combination therapies as an undifferentiated group. Each intervention should instead be matched to a specific, measurable biological barrier, with its effectiveness assessed using the multidimensional endpoint framework described above. Such a barrier-matched approach may reduce reliance on empirical treatment selection and improve the interpretability of trials conducted in the relatively small osteosarcoma patient population. The proposed barrier-matched therapeutic strategies and multidimensional evaluation framework are summarized in **Figure 2**.

**Figure 2:**
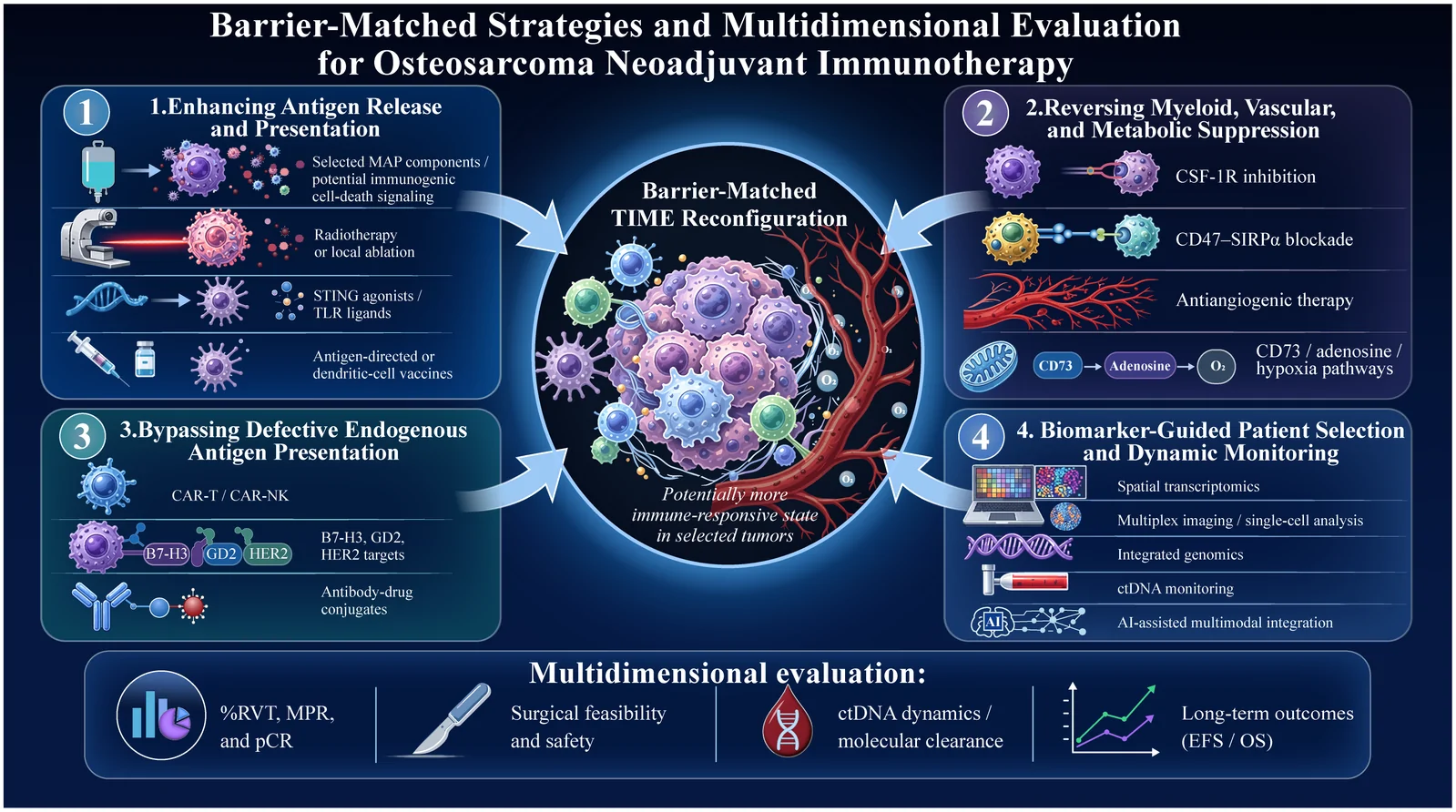
Barrier-matched therapeutic strategies and multidimensional evaluation framework for neoadjuvant immunotherapy in osteosarcoma. This schematic summarizes a barrier-matched framework for the clinical development of neoadjuvant immunotherapy in osteosarcoma. Rather than evaluating heterogeneous combination regimens as an undifferentiated group, each intervention should be selected according to a measurable biological or clinical barrier and assessed using corresponding pharmacodynamic and clinical endpoints. The first strategy aims to enhance antigen release and presentation. Potential approaches include selected components of MAP-based chemotherapy that may promote immunogenic cell-death signaling and modify antigen presentation, radiotherapy or local ablative treatment, intratumoral stimulation of innate immune pathways through STING agonists or Toll-like receptor ligands, and antigen-directed or dendritic-cell-based vaccines. The relevant objective is not antigen release alone, but the generation, processing, and presentation of antigens that can be recognized by functional tumor-reactive lymphocytes. The second strategy seeks to reverse myeloid, vascular, and metabolic immunosuppression. Candidate interventions include colony-stimulating factor 1 receptor inhibition to alter tumor-associated macrophage states, blockade of the CD47–SIRPα axis to enhance macrophage-mediated phagocytosis, antiangiogenic therapy to improve vascular function and reduce hypoxia, and targeting of CD73, adenosine signaling, or hypoxia-associated metabolic pathways. These approaches should be evaluated using pharmacodynamic evidence of pathway modulation rather than changes in immune-cell density alone. The third strategy attempts to bypass defective endogenous HLA-mediated antigen presentation by directly targeting tumor-associated surface antigens. CAR-T-cell and CAR-NK-cell therapies directed against targets such as B7-H3, GD2, or HER2 may provide HLA-independent recognition of tumor-associated surface antigens, whereas antibody–drug conjugates may bypass intracellular antigen processing by selectively delivering cytotoxic payloads to antigen-expressing tumor cells. Their perioperative use requires careful assessment of antigen heterogeneity, treatment-related toxicity, cytopenias, inflammatory complications, and the potential for delay of definitive surgery. The fourth strategy incorporates biomarker-guided patient selection and dynamic monitoring. Spatial transcriptomics, multiplex imaging, single-cell analysis, integrated genomic profiling, circulating tumor DNA monitoring, and artificial intelligence-assisted multimodal integration may help identify immune-desert, immune-excluded, myeloid-dominant, or antigen-presentation-deficient tumors and match patients to specific interventions. The central concept is barrier-matched reconfiguration of the tumor immune microenvironment (TIME), with the aim of shifting selected tumors toward a potentially more immune-responsive state rather than assuming uniform conversion of all osteosarcomas from immunologically “cold” to “hot.” Treatment success should be evaluated multidimensionally using continuous percentage of residual viable tumor (%RVT), major pathological response (MPR), pathological complete response (pCR), completion and timing of planned surgery, R0 resection, perioperative safety, serial circulating tumor DNA dynamics, including molecular clearance when measurable, event-free survival, overall survival, and functional outcomes. No single pathological or molecular endpoint should be regarded as a validated substitute for long-term clinical benefit. ADC, antibody–drug conjugate; AI, artificial intelligence; CAR-NK, chimeric antigen receptor natural killer cell; CAR-T, chimeric antigen receptor T cell; CSF-1R, colony-stimulating factor 1 receptor; ctDNA, circulating tumor DNA; EFS, event-free survival; HLA, human leukocyte antigen; ICI, immune checkpoint inhibitor; MAP, high-dose methotrexate, doxorubicin, and cisplatin; MPR, major pathological response; OS, overall survival; pCR, pathological complete response; SIRPα, signal regulatory protein alpha; STING, stimulator of interferon genes; TIME, tumor immune microenvironment; TLR, Toll-like receptor; %RVT, percentage of residual viable tumor.

### 5.1 Enhancing antigen release and presentation

Chemotherapy remains the most clinically accessible partner for immune checkpoint blockade because selected cytotoxic agents may induce immunogenic cell-death signaling and modulate antigen presentation (19). Future trials should determine which MAP components, dosing schedules, and treatment sequences optimize immune modulation without increasing bone marrow toxicity or delaying definitive surgery. The critical question is not simply whether chemotherapy promotes antigen release, but whether it enhances the presentation of antigens that can be recognized by functional tumor-reactive T-cell clones.

Radiotherapy, local ablative approaches, and oncolytic virotherapy may provide complementary signals for antigen release through immunogenic cell death, and abscopal responses have been reported both clinically after radiotherapy combined with immune checkpoint blockade and preclinically in osteosarcoma (75–77). Their role in osteosarcoma should be investigated cautiously because evidence supporting systemic abscopal effects remains limited, while radiation may impair wound healing or complicate surgical reconstruction. Intratumoral STING agonists and Toll-like receptor (TLR) ligands may enhance type I interferon signaling, dendritic cell activation, and antigen cross-presentation while the primary tumor remains accessible (78, 79). These approaches are more accurately characterized as local reconfiguration of the TIME than as an *in situ* vaccine effect.

Dendritic cell vaccines, personalized antigen-directed vaccines, and vaccine-based combinations with irreversible electroporation are also under investigation (80, 81). A HER2-directed, live-attenuated Listeria-based vaccine has been examined in both dogs and humans, and the two datasets should be read carefully. In dogs with osteosarcoma receiving standard care, immunization did not significantly extend the disease-free interval or overall survival relative to a historical cohort, although the magnitude of the immune response distinguished long-term from short-term survivors (82). The corresponding human study, AOST2121 (NCT04974008), is a single-arm phase 2 trial of the same construct given as maintenance therapy after resection of recurrent, pulmonary-only disease (83). Two constraints limit what can be inferred from it at present: it is an adjuvant rather than a neoadjuvant study, and its outcomes have been reported only through conference and sponsor communications, with no peer-reviewed publication and no results posted to the trial registry. HER2-directed vaccination is therefore best described as a hypothesis under active test rather than an established translational rationale. However, vaccine-induced immune responses should not be assumed to result in pCR or durable disease control in patients with osteosarcoma. Future studies should confirm target expression, HLA-mediated antigen presentation, tumor-reactive T-cell clonality, and correlations between these biological features and pathological and molecular responses.

### 5.2 Reversing myeloid, vascular, and metabolic suppression

Myeloid-directed combination strategies are biologically compelling because tumor-associated macrophages and other suppressive myeloid populations are major components of the osteosarcoma TIME. In sarcoma models, inhibition of colony-stimulating factor 1 receptor (CSF-1R) can reprogram macrophage populations and enhance T-cell infiltration (30). Blockade of the CD47–SIRPα axis may restore macrophage-mediated phagocytosis and enhance antigen cross-presentation (84, 85). These strategies should be evaluated using pharmacodynamic measures of changes in myeloid cell states rather than relying solely on immune cell density.

Antiangiogenic agents may simultaneously improve tumor perfusion, reduce hypoxia, and modify suppressive myeloid and regulatory immune cell populations. The clinical activity observed with apatinib plus camrelizumab and lenvatinib plus pembrolizumab provides a rationale for further investigation (37, 38). However, the dose required to achieve vascular normalization may differ from the maximum tolerated antitumor dose. Perioperative studies must also account for impaired wound healing, bleeding risk, and the optimal timing of treatment discontinuation before surgery.

Metabolic interventions targeting adenosine signaling, CD73, hypoxia-associated pathways, or regulators of ferroptosis may alleviate local immune dysfunction and complement immune checkpoint blockade (1, 31, 86, 87). However, these strategies remain predominantly at the preclinical or early translational stage. Biomarker-enriched studies should therefore confirm that the targeted pathway is active in enrolled tumors and demonstrate that its modulation is associated with reduced %RVT, improved immune function, or enhanced molecular clearance.

### 5.3 Bypassing defective endogenous antigen presentation

Cellular therapies and antibody–drug conjugates may circumvent some limitations of endogenous HLA-mediated antigen presentation by directly targeting cell-surface antigens. B7-H3, GD2, and HER2 are among the most extensively investigated therapeutic targets in osteosarcoma (88–92). Chimeric antigen receptor T-cell (CAR-T) (88–92) and CAR-natural killer cell (CAR-NK) (93) strategies redirect cytotoxicity to surface antigen without requiring endogenous antigen presentation, and therefore offer a means of circumventing the loss or downregulation of HLA class I documented in both primary and metastatic osteosarcoma (23), while natural killer (NK) cells additionally retain major histocompatibility complex (MHC)-independent cytotoxic potential (93). A B7-H3-directed construct has reached the clinic in this population: 3CAR (NCT04897321), a phase 1 dose-escalation trial of autologous B7-H3-specific CAR-T cells in patients aged 21 years or younger with B7-H3-positive solid tumors including osteosarcoma, is recruiting, with safety and the maximum tolerated dose as its primary objective.

Nevertheless, these approaches are limited by antigen heterogeneity, impaired trafficking through the immunosuppressive microenvironment, T-cell exhaustion, cytokine release-associated toxicity, and the risk of on-target, off-tumor effects. Their incorporation into a neoadjuvant treatment pathway requires particular caution because lymphodepletion, prolonged cytopenias, or severe inflammatory toxicities may delay definitive surgery. Early-phase trials should therefore prioritize safety, feasibility, and pharmacodynamic evidence of target engagement before undertaking comparative assessments of pathological response.

B7-H3-directed antibody–drug conjugates provide an alternative approach for selective cytotoxic delivery and may also induce immunogenic cell death. Combining these agents with immune checkpoint blockade is conceptually attractive. However, such combinations should not be described as having proven the conversion of an immunologically “cold” tumor into a “hot” tumor. Trial designs should evaluate the uniformity of target expression, payload-related toxicity, residual viable tumor, surgical complications, and whether any observed immune activation is tumor reactive rather than nonspecific.

### 5.4 Harnessing natural killer cell number and function

NK cells retain cytotoxic potential that does not require HLA class I-mediated presentation, which makes them relevant precisely where the strategies above are constrained, and genotoxic stress shapes the ligands governing that recognition. Chemotherapy-induced genotoxic stress promoted sensitivity to NK-cell cytotoxicity by enabling missing-self recognition, with upregulation of NKG2D ligands but not of class I MHC (94); in human osteosarcoma cell lines, histone deacetylase inhibition increased surface MICA and MICB without increasing their soluble forms (95); and radiotherapy upregulated NKG2D ligands and acted synergistically with NKG2D-directed CAR-T cells in a preclinical glioblastoma model (96). These findings are preclinical, but they identify a hypothesis the neoadjuvant window can test by comparing ligand expression on the resection specimen with that on the pretreatment biopsy.

Two interventions have a defined mechanistic rationale here. Osteosarcoma expresses CD155, which engages the inhibitory receptors TIGIT and CD96 as well as the activating receptor DNAM-1, and in a murine model of pulmonary osteosarcoma metastasis CD155 blockade enhanced the cytotoxicity and degranulation of adoptively transferred allogeneic NK cells in a DNAM-1-dependent manner (97), which is notable for a disease whose dominant metastatic site is the lung. Cytokine-induced memory-like NK cells, preactivated with IL-12, IL-15, and IL-18, show cytotoxicity persisting for weeks (98), although that was demonstrated against myeloid leukemia rather than sarcoma.

The antibody-dependent route has already been tested clinically, and the result is instructive because it was negative. In the Children’s Oncology Group study AOST1421 (NCT02484443), a single-arm phase 2 trial in recurrent pulmonary osteosarcoma in complete surgical remission, dinutuximab with granulocyte-macrophage colony-stimulating factor did not demonstrate efficacy: 11 of 39 eligible patients remained event free, a disease control rate of 28.2% against a historical comparison (99). Target-antigen expression is therefore not sufficient here. Because the trial followed surgical remission rather than preceding planned resection, it could not measure pathological response, leaving this mechanism untested preoperatively rather than excluded.

Preoperative use raises feasibility constraints of its own: cytokine-based expansion risks systemic inflammatory toxicity and adoptive transfer may require lymphodepletion, either of which could delay surgery. Early-phase studies should therefore report time to surgery and pharmacodynamic evidence of NK-cell engagement.

### 5.5 Biomarker-guided patient selection and dynamic monitoring

Spatial transcriptomics, multiplex imaging, single-cell profiling, and integrative genomic analyses can identify immune-desert, immune-excluded, myeloid-dominant, and antigen-presentation-deficient tumor phenotypes, thereby informing barrier-matched treatment selection (100–102). Recent osteosarcoma-specific work has begun to define these phenotypes concretely rather than by analogy with other tumor types. A combined single-cell and spatially resolved atlas of human osteosarcoma has mapped malignant, stromal, and immune compartments together with their spatial relationships within primary tumors (103). Spatial transcriptional profiling of pediatric pulmonary metastases identified a conserved architecture resembling a foreign-body granuloma, characterized by peripheral inflammatory signaling, fibrocollagenous encapsulation, lymphocyte exclusion, and peritumoral macrophage accumulation, whereas the intratumoral compartment lacked inflammatory signaling; the CXCR4 axis emerged from that analysis as a spatially defined candidate target bridging both compartments (104). A subsequent spatial transcriptomic atlas spanning primary tumors and local or metastatic relapses reported systematic lymphoid exclusion, differential myeloid infiltration patterns, and a type I interferon response signature, together with a nine-gene cell-surface signature of theranostic interest (105).

Single-cell profiling adds what spatial data cannot, namely the identity and differentiation state of the cells in each compartment. Sequencing of 100,987 cells from primary, recurrent, and pulmonary metastatic osteosarcoma lesions resolved eleven major cell clusters, inferred transdifferentiation of malignant osteoblastic from malignant chondroblastic cells, and identified a proinflammatory FABP4-expressing macrophage population alongside an immunosuppressive microenvironment in advanced disease (106). The myeloid compartment therefore resolves into distinct populations rather than one suppressive entity, so colony-stimulating factor 1 receptor blockade may deplete one population while sparing another.

These datasets do more than update the descriptive vocabulary of the field. They convert immune exclusion from a qualitative label into a spatially measurable phenotype with named mediators, and they indicate that the dominant barrier may differ between the primary bone lesion and pulmonary metastases within the same patient. That distinction has a direct consequence for trial design: a barrier-matched strategy selected on the basis of a primary-site biopsy may fail to address the barrier operating at the metastatic site that ultimately determines survival. Such approaches are therefore particularly valuable in osteosarcoma, because a limited biopsy specimen may not adequately capture the spatial heterogeneity of the primary tumor or the biological differences between primary and metastatic lesions.

Circulating tumor DNA (ctDNA) provides a complementary longitudinal measure of molecular disease burden and minimal residual disease (67, 69). Serial ctDNA assessment may help clarify equivocal imaging findings, document molecular clearance before surgery, and detect molecular relapse during post-treatment surveillance. However, ctDNA analysis should complement, rather than replace, pathological examination and longitudinal clinical follow-up.

Deep learning applied to osteosarcoma histology images has been explored for predicting chemotherapy response (107), and the wider promise of artificial intelligence lies in integrating clinical, imaging, pathological, and omics data within a single model. However, predictive models require external validation, transparent definition of input features, and appropriate calibration in prospectively assembled osteosarcoma cohorts. The neoadjuvant treatment window provides an efficient setting for such investigations because pretreatment biopsy specimens, serial blood samples, longitudinal imaging data, and post-treatment resection specimens can be integrated at the individual-patient level.

Beyond their role in staging, the TDLNs are a site of antigen presentation and T-cell priming, and this has prompted proposals that lymphatic preservation be weighed during perioperative treatment planning (108). Preservation of lymphatic drainage and TDLN function during the preoperative treatment interval may support coordinated antitumor immune responses. However, this hypothesis does not justify modifying or compromising oncologically indicated surgery. Prospective studies should instead determine how treatment timing and the extent of lymph-node intervention affect immune activation, pathological response, and long-term disease control.

## 6 Conclusion

Neoadjuvant immunotherapy represents a potential strategy for overcoming the long-standing therapeutic plateau in osteosarcoma. Administering treatment while the primary tumor, lymphatic drainage, and TDLNs remain intact may provide an opportunity to reconfigure the TIME, enhance antigen presentation, and promote the recruitment, priming, or functional reinvigoration of tumor-reactive T-cell clones. However, this model should not be equated with vaccination. TILs are heterogeneous and may include bystander or virus-specific cells. Therefore, increased lymphocyte infiltration or expansion does not necessarily indicate tumor-specific reactivity, durable immune memory, or effective antitumor protection. Any systemic control of occult micrometastatic disease will depend on the generation of functionally competent tumor-reactive immunity and remains to be demonstrated prospectively in osteosarcoma.

The generally low yet heterogeneous immunogenicity of osteosarcoma, together with defective antigen presentation, myeloid cell dominance, immune exclusion, and metabolic and stromal barriers, limits the effectiveness of immune checkpoint inhibitor monotherapy. Future clinical development should prioritize rational combination strategies matched to clearly defined biological barriers. Such strategies should be evaluated for their ability to shift selected tumors toward a more immune-responsive state rather than being assumed to uniformly convert all osteosarcomas from immunologically “cold” to “hot.” Structural genomic instability modifies which barrier predominates rather than predicting benefit, and HLA-independent strategies, including natural killer cell-directed approaches, merit parallel evaluation.

The clinical scope of neoadjuvant immunotherapy should be defined according to treatment history, disease extent, and resectability. Initial clinical development should prioritize treatment-naive patients with localized, resectable disease, for whom the surgical pathway and pathological response endpoints are most clearly defined. Previously treated patients with resectable recurrence or oligometastatic disease may be evaluated in separately designed perioperative or preoperative salvage cohorts, provided that investigational treatment does not delay or compromise complete surgical clearance. Achievement of a second complete surgical remission or complete metastasectomy remains a major determinant of long-term survival in appropriately selected patients with recurrent or pulmonary metastatic disease (46, 47). In this setting, immunotherapy should therefore be investigated as an adjunct to complete local control rather than as a substitute for it.

Pathological response should be positioned as a central but currently unvalidated early endpoint. Continuous %RVT, MPR, and pCR should be interpreted in conjunction with completion of the planned surgery, achievement of R0 resection, perioperative morbidity, circulating tumor DNA clearance, event-free survival, overall survival, and functional outcomes. The neoadjuvant treatment window also provides a valuable translational platform for paired assessment of antigen presentation, T-cell clonality and specificity, spatial immune organization, and treatment-induced remodeling of the TIME.

Future trials should evaluate treatment-naive localized disease, previously treated recurrence, resectable oligometastatic disease, and unresectable disseminated disease in separate and appropriately stratified cohorts. Multicenter collaboration among orthopedic oncologists, medical oncologists, pathologists, radiologists, and translational immunologists will be essential to determine whether biomarker-guided, barrier-matched neoadjuvant or perioperative immunotherapy can safely induce clinically meaningful pathological responses and improve long-term outcomes in selected patients with osteosarcoma.

## Supplementary material

Supplementary Figure 1 Conceptual evolution of immunotherapy strategies in osteosarcoma from checkpoint monotherapy to barrier-matched neoadjuvant and perioperative approaches. This figure presents a conceptual, rather than strictly chronological, framework for the development of immunotherapy strategies in osteosarcoma. The developmental phases do not represent formal clinical stages or an established hierarchy of efficacy. Developmental Phase 1 represents immune checkpoint inhibitor monotherapy evaluated predominantly in patients with advanced, recurrent, refractory, or metastatic osteosarcoma. Studies such as SARC028, KEYNOTE-051, and the phase 2 pembrolizumab trial conducted by Boye et al. demonstrated the feasibility of PD-1/PD-L1 pathway inhibition but reported generally low objective response rates. These trials were conducted mainly in salvage settings and did not incorporate prospectively planned definitive surgery or standardized pathological-response assessment. Consequently, they provide information regarding drug activity and resistance but do not establish neoadjuvant efficacy. Developmental Phase 2 represents combination strategies, particularly antiangiogenic agents combined with immune checkpoint blockade. Apatinib plus camrelizumab and lenvatinib plus pembrolizumab have produced signals of greater antitumor activity in selected advanced sarcoma or osteosarcoma cohorts. Antiangiogenic agents may contribute through vascular normalization, reduction of hypoxia, and partial modification of suppressive myeloid or regulatory-cell populations. However, the available evidence remains derived largely from advanced or salvage-treatment cohorts and does not demonstrate improved major pathological response, pathological complete response, resectability, perioperative safety, or postoperative outcomes. Potential increases in overlapping toxicity must also be considered. Developmental Phase 3 represents future barrier-matched neoadjuvant or perioperative strategies. These approaches may involve chemotherapy and/or immune checkpoint inhibition, with or without additional interventions selected according to specific antigen-presentation, myeloid, vascular, metabolic, stromal, or biomarker-defined barriers. Such strategies require prospective evaluation within clearly defined surgical pathways. Key endpoints should include continuous %RVT, MPR, pCR, completion and timing of planned surgery, R0 resection or complete metastasectomy, perioperative morbidity, serial circulating tumor DNA dynamics, including molecular clearance when measurable, event-free survival, overall survival, and functional outcomes. The figure does not imply that combination therapy has already improved pathological response or survival in resectable osteosarcoma; rather, it illustrates a proposed direction for biomarker-guided clinical development. ctDNA, circulating tumor DNA; ICI, immune checkpoint inhibitor; MAP, high-dose methotrexate, doxorubicin, and cisplatin; MPR, major pathological response; pCR, pathological complete response; PD-1, programmed cell death protein 1; PD-L1, programmed death-ligand 1; %RVT, percentage of residual viable tumor.
